# Association between exposure to metalworking fluid aerosols, occupational noise and chronic kidney disease: a cross-sectional study in China

**DOI:** 10.1186/s12889-024-19006-7

**Published:** 2024-06-04

**Authors:** Li Zhou, Beining Wu, Minzhu Tang, Geyang Li, Weiling Chan, Lin Song, Jin Wang, Lejia Zhu, Lan Lin, Yulong Lian

**Affiliations:** 1 Department of Epidemiology and Medical Statistics, School of Public Health, Nantong University, Se Yuan Road, No. 9, Nantong, Jiangsu 226001 China; 2 Wuxi Eighth People’s Hospital, Wuxi, Jiangsu China

**Keywords:** Metalworking fluid aerosols, Occupational noise exposure, CKD, Interaction, Threshold effect

## Abstract

**Background:**

Chronic kidney disease (CKD) carries a high public health burden yet little is known about the relationship between metalworking fluid (MWF) aerosols, occupational noise and CKD. We aimed to explore the relationship between occupational MWF aerosols, occupational noise and CKD.

**Methods:**

A total of 2,738 machinists were sampled from three machining companies in Wuxi, China, in 2022. We used the National Institute for Occupational Safety and Health (NIOSH) method 5524 to collect individual samples for MWF aerosols exposure, and the Chinese national standard (GBZ/T 189.8–2007) method to test individual occupational noise exposure. The diagnostic criteria for CKD were urinary albumin/creatinine ratio (UACR) of ≥ 30 mg/g and reduced renal function (eGFR < 60 mL.min^− 1^. 1.73 m^− 2^) lasting longer than 3 months. Smooth curve fitting was conducted to analyze the associations of MWF aerosols and occupational noise with CKD. A segmented regression model was used to analyze the threshold effects.

**Results:**

Workers exposed to MWF aerosols (odds ratio [*OR*] = 2.03, 95% confidence interval [*CI*]: 1.21–3.41) and occupational noise (*OR* = 1.77, 95%*CI*: 1.06–2.96) had higher prevalence of CKD than nonexposed workers. A nonlinear and positive association was found between increasing MWF aerosols and occupational noise dose and the risk of CKD. When daily cumulative exposure dose of MWF aerosols exceeded 8.03 mg/m^3^, the *OR* was 1.24 (95%*CI*: 1.03–1.58), and when occupational noise exceeded 87.22 dB(A), the *OR* was 1.16 (95%*CI*: 1.04–1.20). In the interactive analysis between MWF aerosols and occupational noise, the workers exposed to both MWF aerosols (cumulative exposure ≥ 8.03 mg/m^3^-day) and occupational noise (L_EX,8 h_ ≥ 87.22 dB(A)) had an increased prevalence of CKD (*OR* = 2.71, 95%*CI*: 1.48–4.96). MWF aerosols and occupational noise had a positive interaction in prevalence of CKD.

**Conclusions:**

Occupational MWF aerosols and noise were positively and nonlinearly associated with CKD, and cumulative MWF aerosols and noise exposure showed a positive interaction with CKD. These findings emphasize the importance of assessing kidney function of workers exposed to MWF aerosols and occupational noise. Prospective and longitudinal cohort studies are necessary to elucidate the causality of these associations.

**Supplementary Information:**

The online version contains supplementary material available at 10.1186/s12889-024-19006-7.

## Background

Chronic kidney disease (CKD) affects approximately 10% of the world’s population and is associated with substantial morbidity and mortality [1]. According to research published in the *Lancet* in 2020, the global total age mortality rate for CKD increased by 41.5% over the previous 27 years, accounting for 1.4 million cardiovascular disease–related deaths and 25.3 million disability-adjusted life years of cardiovascular disease attributed to impaired kidney function, bringing a significant burden to healthcare systems around the world [2]. The prevalence of CKD is 10.8% nationwide, affecting approximately 120 million patients according to the China Cardiovascular Disease Report (2021) [3]. Early detection and intervention can raise the level of prevention and treatment of CKD, enhance the prognosis, lessen the associated medical and financial burden, and delay, halt, or even reverse the development of CKD [4].

Metalworking fluid (MWF) and occupational noise are common occupational pollutants in machining industry worldwide. MWF aerosols include all contaminants in mists and fogs generated during metals and metal-substitute grinding and machining operations. In the United States, over 1 billion workers are exposed to MWFs every day at work [5]. Additionally, according to Colbeth et al., the size of the global market for MWFs is expected to reach $16 billion by 2027 [6]. China produced 601,700 units of metal-cutting machine tools in 2021 [7]. The increasing use of computer numerical control (CNC) machine tools has raised the need for MWF. The National Institute for Occupational Safety and Health (NIOSH) recommends that exposure to MWF aerosols should be limited to 0.5 milligrams (mg) per cubic meter of air (total particulate mass) as a time-weighted average of concentrations for a 40-hour workweek or 10-hour workday [8]. Although studies on occupational exposure limits for MWF have been published [8, 9], there are no set thresholds for cumulative exposure. Shrestha et al. have supposed that long-term exposure to MWF aerosols correlates with kidney cancer and glomerulonephritis [10, 11]. According to previous research, MWF aerosols contain sulfonates, nonionic surfactants, ethanolamine, medium-chained chlorinated paraffins (MCCPs), and polycyclic aromatic hydrocarbons (PAHs) [6]. Zhao et al. demonstrated that exposure to PAHs activates the aryl hydrocarbon receptor (AhR) signaling pathway [12], promoting the occurrence and further development of CKD. Diethanolamines, PAHs, and MCCPs have all been shown in numerous animal studies to induce glomerular dysfunction and interfere with normal renal metabolism [13–16]. Thus, there might be a connection between MWF aerosols and CKD.

Occupational noise is also one of the most common exposures in machining industry fields [17]. Metal impact is a substantial source of noise in the production area. Specifically, metal conduit rolling into or dropping onto other pieces of conduit during processing causes impact noise. The production equipment itself also generates noise when it is running. According to Angel et al., environmental noise is associated with decreased glomerular filtration rate [18], which may lead to a higher prevalence of CKD. The concrete molecular mechanism may be as follows: occupational noise activates the neuroendocrine hypothalamus–pituitary–adrenocortical cortex axis, thereby producing corticotropin-releasing hormone and noradrenaline [19]. This process may lead to oxidative stress, cause vascular damage [20], alter renal hemodynamics and metabolism, and promote the occurrence and development of CKD. The above studies suggest that noise exposure may be associated with the prevalence of CKD. Currently, the recommended exposure limit on hearing is 85 dB(A), as an 8-hour time-weighted average [21]. However, there is lack of thresholds for other organs. There has been no population-based epidemiological study indicating the relationship between MWF aerosols and CKD, nor there is any research supporting the synergistic effect of MWF and occupational noise on CKD.

Based on the previous studies, it is evident that more research is required to explore the relationship among MWF aerosols, occupational noise, and CKD, and estimate threshold for MWF aerosols and occupational noise. Therefore, in this cross-sectional study, we aimed to investigate whether MWF aerosols, occupational noise, and their synergy are associated with CKD in Chinese machining industry workers. For these analyses, we proposed the following hypotheses: (1) occupational exposure to MWF aerosols correlates with CKD in machinists; (2) occupational noise exposure is associated with the prevalence of CKD in machinists; (3) there is a positive interaction between exposure to occupational MWF aerosols and occupational noise on CKD.

## Materials and methods

### Participants and procedures

From June to August 2022, we conducted a cross-sectional study of employees working in three automobile machining enterprises in Wuxi City, Jiangsu Province. Multi-stage sampling method was used in this study, and the sampling strategy was as follows: Three companies were randomly selected from the seven automotive machinery processing companies in Wuxi City, including one representative of large-sized, medium-sized, and small-sized companies. Employees were randomly selected to participate in the study according to the number of employees in these companies. A total of group of 2,830 individuals sampled: 1,400 individuals were sampled from large enterprises (≥ 3,000 employees), 1,200 from medium-sized enterprises (between 500 and 3,000 employees), and 230 from small enterprises (< 500 employees). The employees uniformly had occupational health examinations at the branch of the Wuxi Eighth People’s Hospital, and all the participants signed a consent form after being informed about the study. The study was approved by the Ethics Committee of Nantong University (2013-L073). The sample size was calculated using the following formula:

$$n=\frac{{Z}_{1-\alpha /2}^{2}\times pq}{{d}^{2}},$$

where α = 0.05, *p* = 0.108 (according to the China Cardiovascular Health and Disease Report 2021, the prevalence of CKD is 10.8% [3]), q = 1 − p, and d = 0.15p. According to the formula, the final sample size required 1,468 active employees.

To improve the accuracy of the results, we used specific inclusion and exclusion criteria to further select the subjects. The inclusion criteria were as follows: (1) 1 year or more in service; (2) willingness to accept occupational health examination and custom questionnaire survey. The exclusion criteria were as follows: (1) employed for less than 1 year (*n* = 35); (2) pregnancy (*n* = 2); (3) a history of previous solid organ transplantation (*n* = 1); (4) a recent history of taking cephalosporins, aminoglycoside antibiotics, flucytosine, cisplatin, cimetidine, trimethoprim, or other drugs (*n* = 22); (5) the response rate of the questionnaire less than 80% or refusing occupational health examinations (*n* = 32). There were no significant differences in the general demographic characteristics of responders and nonresponders (Additional file 1: Table S1). Finally, 2,738 on-the-job workers were included in the study. The participants mainly included the following types of work: numerical control machining center (CNC) operators, CNC maintenance workers, material transporters, sandblasters, anode oxygenators, managers, and security guards.

### MWF aerosols assessment

To ensure the validity and authenticity of the measurements, occupational exposure to MWF aerosols of the workers in the CNC workshops was measured in line with the NIOSH Metalworking Fluids All Categories Method 5524 [22]. In accordance with the Chinese specifications of air sampling for hazardous substances monitoring in the workplace (GBZ.159–2004), the workers were randomly selected for individual sampling according to the number of workers in each type of occupational category. Namely, when the number of workers in each occupation type was < 3, all of them were chosen as sampling objects; when the number of workers was between 3 and 5, two individuals were randomly selected for sampling; when the number of workers was between 6 and 10, three individuals were randomly selected; when the number of workers was > 10, four individuals were randomly selected [23]. Ultimately 52 workers were selected for sampling. Each individual sampling pump was calibrated with a representative calibrator prior to sampling. The personal sampling pump was worn on the worker’s chest in the breathing zone approximately 25 cm from the nose, and sampling was performed at a flow rate of 2 L/minute on three consecutive days between 8:00 a.m. and 4:00 p.m. We calculated the 8-hour time-weighted average (8-h TWA) concentration in accordance with the Specifications of air sampling for hazardous substances monitoring in the workplace GBZ.159–2004 as follows [23]:

$${C}_{TWA}=\frac{c\times v}{F\times 480}\times 1000,$$

where C_TWA_ represents 8-h TWA (mg/m^3^), c represents concentration of harmful substances in sample solution (µg/mL), v represents total volume of sample solution (mL), and F represents sampling flow rate (mL/minute). A job exposure matrix (JEM) [24] for MWF aerosols was used, which combined sampling data with workshop distribution, occupational category, workflow, historical workshop occupational monitoring data, and consultation with local industrial hygienists to fully assess the exposure of each worker. To further analyze the impact of exposure, we divided MWF aerosols exposure into four groups based on the interquartile spacing of the 8-h TWA of each worker for the discussion: 0 < 8-h TWA ≤ 0.39 mg/m^3^; 0.40 mg/m^3^ < 8-h TWA ≤ 0.45 mg/m^3^; 0.46 mg/m^3^ < 8-h TWA ≤ 0.48 mg/m^3^; and 0.49 mg/m^3^ < 8-h TWA ≤ 1.40 mg/m^3^.

The following formula was used to calculate the cumulative exposure dose [25]:

$$C={C}_{TWA}\times \sum _{j=1}^{n}Tj,$$

where C represents the daily cumulative exposure dose (mg/m^3^), C_TWA_ represents 8-h TWA, and T_j_ is the number of hours of work in the corresponding position per day.

### Occupational noise assessment

Individual noise exposure sampling of the workers using personal noise dosimeters and selecting workers were performed in accordance with the Chinese measurement of physical factors in the workplace Part 8: Noise GBZ/T 189.8–2007 [26]. The workers were randomly selected for individual sampling according to the number of workers in each type of occupational category the workers were randomly selected for individual sampling according to the number of workers in each type of occupational category. Namely, when the number of workers in each occupation type was < 3, all of them were chosen as sampling objects; when the number of workers was between 3 and 5, two individuals were randomly selected for sampling; when the number of workers was between 6 and 10, three individuals were randomly selected; when the number of workers was > 10, four individuals were randomly selected [26]. Ultimately 33 workers were selected for sampling. The sampling equipment included sound level calibrator HS6020 Level-SD-1137 and personal sound exposure meter ASV5910. The personal noise dosimeter was set to A-weighting and slow gear, and took the value of the equivalent sound level L_Aeq_. The personal sound exposure meter was calibrated to an error of less than 0.5 dB(A) with a calibrator before each measurement. The personal sound exposure meter was worn on the worker’s front chest about 10 cm from the external ear canal during the measurements. Samples were taken on three consecutive days between 8:00 a.m. and 4:00 p.m. The 8-hour equivalent sound level was calculated, and the average of the three measurements was used as the worker’s noise exposure level. We recorded the measurement details, including workshops, date, position, operating procedure, and measurement duration. We calculated the 8-hour equivalent sound level using the following equation [26]:

$${L}_{EX,8h}={L}_{Aeq,{T}_{e}}+10\text{log}\frac{{T}_{e}}{{T}_{0}},$$

where L_EX,8 h_ is normalization of equivalent continuous A-weighted sound pressure level to a normal 8 h working day, expressed in dB(A); T_e_ is the actual working time of a working day, expressed in hours; L_Aeq, Te_ is the equivalent sound level of the actual working day, expressed in dB(A); and T_0_ is the standard working time (8 h). We used a JEM [24] for occupational noise, which combined sampling data with workshop distribution, occupational category, workflow, historical workshop occupational monitoring data, and equipment replacement to fully assess the exposure of each worker. To further analyze the impact of noise exposure, we divided occupational noise exposure into four groups based on the interquartile spacing of the L_EX,8 h_ for the discussion: 0 < L_EX,8 h_ ≤ 84.85 dB(A); 84.86 dB(A) < L_EX,8 h_ ≤ 89.70 dB(A); 89.71 dB(A) < L_EX,8 h_ ≤ 91.96 dB(A); and 91.97 dB(A) < L_EX,8 h_ ≤ 105.19 dB(A).

### CKD

Morning fasting venous blood and morning urine were uniformly collected from the study participants at the Affiliated Branch of Wuxi Eighth People’s Hospital on the day of physical examination. After centrifugation of the blood samples in the hospital’s laboratory department, the samples were tested for serum creatinine using the Direxion CS-100 automatic biochemistry analyzer; and urine albumin and urine creatinine were tested in the hospital’s laboratory using the automated urine microalbumin creatinine analyzer ACR-300. The corresponding eGFR was calculated using the Chronic Kidney Disease Epidemiology Collaboration 2021 (CKD-EPI) creatinine estimation eGFR equation [27]. The diagnostic criteria for CKD were urinary albumin/creatinine ratio (UACR) of ≥ 30 mg/g and reduced renal function (eGFR < 60 mL.min^− 1^. 1.73 m^− 2^) lasting longer than 3 months, in line with the Guidelines for the Chinese Early Evaluation and Management of Chronic Kidney Disease [28].

### Covariates

We used a self-administered questionnaire to collect covariates, which consisted mainly of basic demographic characteristics (sex, age, body mass index [BMI], ethnicity, marital status), occupational history (use of protective equipment [masks and earplugs], length of service), and risk factors considered to be related to renal function (physical exercise, smoking, drinking, hypertension, diabetes, and family history of kidney disease) [29].

Sex was categorized as male and female. Age was categorized as less than 30 years, 30 to 40 years, 40 to 50 years, and 50 years and older. The use of protective equipment (masks and earplugs) was categorized as yes and no. The ethnicity was divided into Han and ethnic minorities. Marital status was classified as unmarried, married, and divorced. The length of service was categorized as less than 5 years, 5 to 10 years, and more than 10 years. The family history of kidney disease was categorized as yes and no. Referring to the classification of BMI by the China Obesity Working Group, BMI was categorized as below 24 kg/m^2^ and 24 kg/m^2^ and above [30]. The individual’s physical activity level was divided into three groups, namely high, moderate, and low [25]. Patients diagnosed with hypertension in accordance with the Chinese Guidelines for the Prevention and Treatment of Hypertension 2018 were categorized as yes and no [31]. In line with the Chinese Diabetes Diagnostic Guidelines 2022, participants were classified as diabetes and non-diabetes [32]. Smoking was classified as nonsmoking, occasional smoking (smoking less than one cigarette per day but at least four cigarettes per week), and frequent smoking (smoking at least one cigarette per day for 6 months or more) [33]. Drinking was classified as no alcohol, occasional drinking (drinking less than once a week), and regular drinking (drinking at least once a week for 6 months or more) [33].

### Statistical analysis

SPSS 26 and R 4.2 were used for data analysis. Descriptive statistical methods were used to describe the distribution of general demographic characteristics. The composition ratio of count data was analyzed by the chi-square test. Binary logistic regression was used to analyze the relationship among occupational MWF aerosols, noise exposure, and prevalence of CKD. Smooth curve fitting [34] was used to evaluate the potential nonlinear relationships of MWF aerosols and occupational noise with CKD. A segmented regression model was used to analyze the threshold effect, based on the assumption that prevalence of by MWF aerosols and occupational noise is a threshold response, one potential breakpoint was assumed [35]. Model 1 was not adjusted for any confounders. Model 2 was adjusted for physical exercise, smoking and drinking (the factors with statistically significant differences in univariate analyses). The Delta method introduced by Hosmer and Lemeshow [36] was used to estimate the confidence intervals of relative excess risk of interaction (*RERI*), attributable proportion of interaction (*AP*), and synergy index (*S*), to determine whether there was a specific interaction between MWF aerosols exposure and noise exposure on CKD in workers. In this study, bilateral statistical tests were used, and the test level was α = 0.05.

### Ethical considerations

All the participants signed an informed consent form after learning about research-related information. The study was approved by the Ethics Committee of Nantong University (2013-L073).

## Results

A total of 2,738 study participants were included in the study, the prevalence of CKD was 2.74%. Their baseline characteristics are shown in Table 1. Sex, age, marital status, BMI, physical exercise, smoking, drinking, use of protective equipment, length of service, hypertension, and diabetes were significantly different between the groups exposed and not exposed to MWF aerosols (*P* < 0.05). Sex, BMI, physical exercise, smoking, use of protective equipment, length of service, and diabetes were also significantly different between the groups exposed and not exposed to noise (*P* < 0.05). Table 2 shows the results of the univariate analysis of CKD. Compared with individuals with a high level of physical exercise, those with a low level of physical exercise had a higher prevalence of CKD (*OR* = 2.12, 95% *CI*: 1.09–4.12). Regular smokers had a higher prevalence of CKD than nonsmokers (*OR* = 2.08, 95% *CI*: 1.11–3.91). Frequently drinkers had a higher prevalence of CKD than nondrinkers (*OR* = 1.99, 95% *CI*: 1.06–9.55). The prevalence of CKD showed no significant differences in relation to sex, age, ethnicity, marital status, BMI, use of protective equipment, length of service, hypertension, diabetes, and family history of kidney disease (*P* > 0.05).

**Table 1:** Comparison between different demographic characteristics

Variable	Total (2,738)	MWF aerosols		*P*	Noise		*P*
		Nonexposed (1,151)	Exposed (1,587)		Nonexposed (1,170)	Exposed (1,568)	
Sex							
Male	2,288	818 (71.07)	1,470 (92.63)	< 0.01	910 (77.78)	1,378 (87.88)	< 0.01
Female	450	333 (28.93)	117 (7.37)		260 (22.22)	190 (12.12)	
Age							
< 30	854	163 (14.16)	691 (43.54)	< 0.01	366 (31.28)	488 (31.12)	0.96
30–40	1,023	542 (47.09)	481 (30.31)		434 (37.09)	589 (37.56)	
40–50	578	351 (30.50)	227 (14.30)		245 (20.94)	333 (21.24)	
≥ 50	283	95 (8.25)	188 (11.85)		125 (10.68)	158 (10.08)	
Nation							
Han	2,719	1,140 (99.04)	1,579 (99.50)	0.16	1,163 (99.40)	1,556 (99.23)	0.60
Minority	19	11 (0.96)	8 (0.50)		7 (0.60)	12 (0.77)	
Marital status							
Unmarried	752	115 (9.99)	637 (40.14)	< 0.01	317 (27.09)	435 (27.74)	0.51
Married	1,966	1,023 (88.88)	943 (59.42)		842 (71.97)	1,124 (71.68)	
Divorced	20	13 (1.13)	7 (0.44)		11 (0.94)	9 (0.57)	
BMI							
< 24 mg/m^2^	2,530	999 (86.79)	1,531 (96.47)	< 0.01	1,008 (86.15)	1,522 (97.07)	< 0.01
≥ 24 mg/m^2^	208	152 (13.11)	56 (3.53)		162 (13.85)	46 (2.93)	
Physical exercise							
High	346	178 (15.46)	168 (10.59)	< 0.01	169 (14.44)	177 (11.29)	< 0.01
Moderate	596	299 (25.98)	297 (18.71)		356 (30.43)	240 (15.31)	
Low	1,796	674 (58.56)	1,122 (70.70)		645 (55.13)	1,151 (73.40)	
Smoking							
Never	1,526	726 (63.08)	800 (50.41)	< 0.01	687 (58.72)	839 (53.51)	< 0.01
Occasionally	526	101 (8.77)	425 (26.78)		238 (20.34)	288 (18.37)	
Frequently	686	324 (28.15)	362 (22.81)		245 (20.94)	441 (28.12)	
Drinking							
Never	1,695	762 (66.20)	933 (58.79)	< 0.01	742 (63.42)	953 (60.78)	0.25
Occasionally	1,019	384 (33.36)	635 (40.01)		416 (35.56)	603 (38.46)	
Frequently	24	5 (0.43)	19 (1.20)		12 (1.02)	12 (0.76)	
Use of protective equipment							
Yes	2,624	1,127 (97.91)	1,497 (94.33)	< 0.01	1,141 (97.52)	1,483 (94.58)	< 0.01
No	114	24 (2.09)	90 (5.67)		29 (2.48)	85 (5.42)	
Length of service							
1–5 years	2,337	1,148 (99.74)	1,189 (74.92)	< 0.01	1,051 (89.83)	1,286 (82.02)	< 0.01
5–10 years	250	1 (0.09)	249 (15.67)		77 (6.58)	173 (11.03)	
≥ 10 years	151	2 (0.17)	149 (9.39)		42 (3.59)	109 (6.95)	
Hypertension							
No	2,480	1,001 (86.97)	1,479 (93.19)	< 0.01	1,069 (91.37)	1,411 (89.99)	0.22
Yes	258	150 (13.03)	108 (6.81)		101 (8.63)	157 (10.01)	
Diabetes							
No	2,002	773 (67.16)	1,229 (77.44)	< 0.01	826 (70.60)	1,176 (75.00)	0.01
Yes	736	378 (32.84)	358 (22.56)		344 (29.40)	392 (25.00)	
Family history of CKD							
No	2,696	1,129 (98.09)	1,567 (98.74)	0.17	1,148 (98.12)	1,548 (98.72)	0.20
Yes	42	22 (1.91)	20 (1.26)		22 (1.88)	20 (1.28)	

**Table 2:** Univariate analysis of CKD

Variables	Case/Prevalence rate	OR (95% CI)	*P*
Sex			
Male	63 (2.75)	1.00	
Female	12 (2.67)	1.73 (0.87–3.86)	0.14
Age			
< 30	18 (2.11)	1.00	
30–40	25 (2.44)	1.20 (0.62–2.32)	0.59
40–50	20 (3.46)	1.82 (0.88–3.76)	0.11
≥ 50	12 (4.24)	2.02 (0.94–4.36)	0.07
Nation			
Han	75 (2.76)	1.00	
Minority	0 (0)	—	—
Marital status			
Unmarried	23 (3.06)	1.00	
Married	51 (2.59)	1.01 (0.60–1.72)	0.96
Divorced	1 (5.00)	2.75 (0.34–22.24)	0.34
BMI			
< 24 mg/m^2^	12 (4.07)	1.00	
≥ 24 mg/m^2^	63 (5.77)	1.07 (0.79–1.47)	0.66
Physical exercise			
High	10 (2.89)	1.00	
Moderate	28 (4.70)	1.75 (0.89–3.50)	0.11
Low	37 (5.51)	2.12 (1.09–4.12)	0.03
Smoking			
Never	31 (2.03)	1.00	
Occasionally	19 (3.61)	1.83 (0.90–3.70)	0.10
Frequently	25 (3.64)	2.08 (1.11–3.91)	0.02
Drinking			
Never	43 (2.54)	1.00	
Occasionally	30 (2.94)	0.94 (0.54–1.64)	0.84
Frequently	2 (8.33)	1.99 (1.06–9.55)	0.04
Use of protective equipment			
Yes	68 (4.99)	1.00	
No	7 (6.14)	1.78 (0.82–7.58)	0.12
Length of service			
1–5 years	57 (2.44)	1.00	
5–10 years	9 (3.60)	1.12 (0.51–2.42)	0.78
≥ 10 years	9 (5.96)	1.56 (0.92–3.40)	0.13
Hypertension			
No	68 (2.74)	1.00	
Yes	7 (2.71)	0.92 (0.40–2.09)	0.84
Diabetes			
No	58 (2.90)	1.00	
Yes	17 (2.31)	0.84 (0.48–1.49)	0.56
Family history of CKD			
No	74 (2.74)	1.00	
Yes	1 (2.38)	0.86 (0.12–6.37)	0.89

Table 3 shows the associations of exposure to MWF aerosols and occupational noise with the prevalence of CKD. A positive relationship was found between MWF aerosols exposure and CKD prevalence (*OR* = 2.12, 95% *CI*: 1.15–3.92). When cumulative MWF aerosols exposure was classified as a quintile categorical variable, using the first quintile as a reference, the *OR*s (95% *CI*) of CKD after adjustment for physical exercise, smoking and drinking were 1.00, 1.23 (0.50–2.99), 1.86 (0.85–4.08), and 2.23 (1.01–4.95) across increasing quintiles of cumulative MWF aerosols exposure. A positive relationship was also found between occupational noise exposure and CKD prevalence (*OR* = 1.83, 95% *CI*: 1.10–3.02). When occupational noise exposure was classified as a quintile categorical variable, using the first quintile as a reference, the *OR*s (95% *CI*) of CKD after full adjustment for physical exercise, smoking and drinking were 1.00, 1.38 (0.50–3.11), 2.36 (1.23–5.38), and 2.66 (1.18–6.03) across increasing noise quintiles.

**Table 3:** Logistic regression analysis of occupational MWF aerosols exposure, noise exposure, and prevalence of CKD

Variables	Case/Prevalence	Model 1		Model 2	
		OR	95% CI	OR	95% CI
MWF aerosols (mg/m^3^)					
Nonexposed	20 (1.7)	1.00		1.00	
Exposed	55 (3.5)	2.12	(1.15–3.92) ^*^	2.03	(1.21–3.41) ^*^
Cumulative exposure dose (mg/m^3^-day)					
< 0-0.39	10 (2.2)	1.00		1.00	
0.4–0.45	10 (2.8)	1.28	(0.53–3.12)	1.23	(0.50–2.99)
0.46–0.48	18 (4.1)	1.89	(0.86–4.14)	1.86	(0.85–4.08)
0.49–1.40	17 (5.0)	2.34	(1.06–5.18) ^*^	2.23	(1.01–4.95) ^*^
Noise [dB(A)]					
Nonexposed	22 (1.9)	1.00		1.00	
Exposed	53 (3.4)	1.83	(1.10–3.02) ^*^	1.77	(1.06–2.96) ^*^
< 0-84.85	9 (2.0)	1.00		1.00	
84.86–89.70	9 (2.7)	1.32	(0.57–3.90)	1.38	(0.50–3.11)
89.71–91.96	18 (4.3)	2.16	(1.01–4.87) ^*^	2.36	(1.23–5.38) ^*^
91.97-105.19	18 (5.0)	2.54	(1.13–5.73) ^*^	2.66	(1.18–6.03) ^*^

Model 1: unadjusted; Model 2: Adjusted for physical exercise, smoking and drinking; ^*^P < 0.05

**Fig. 1:**
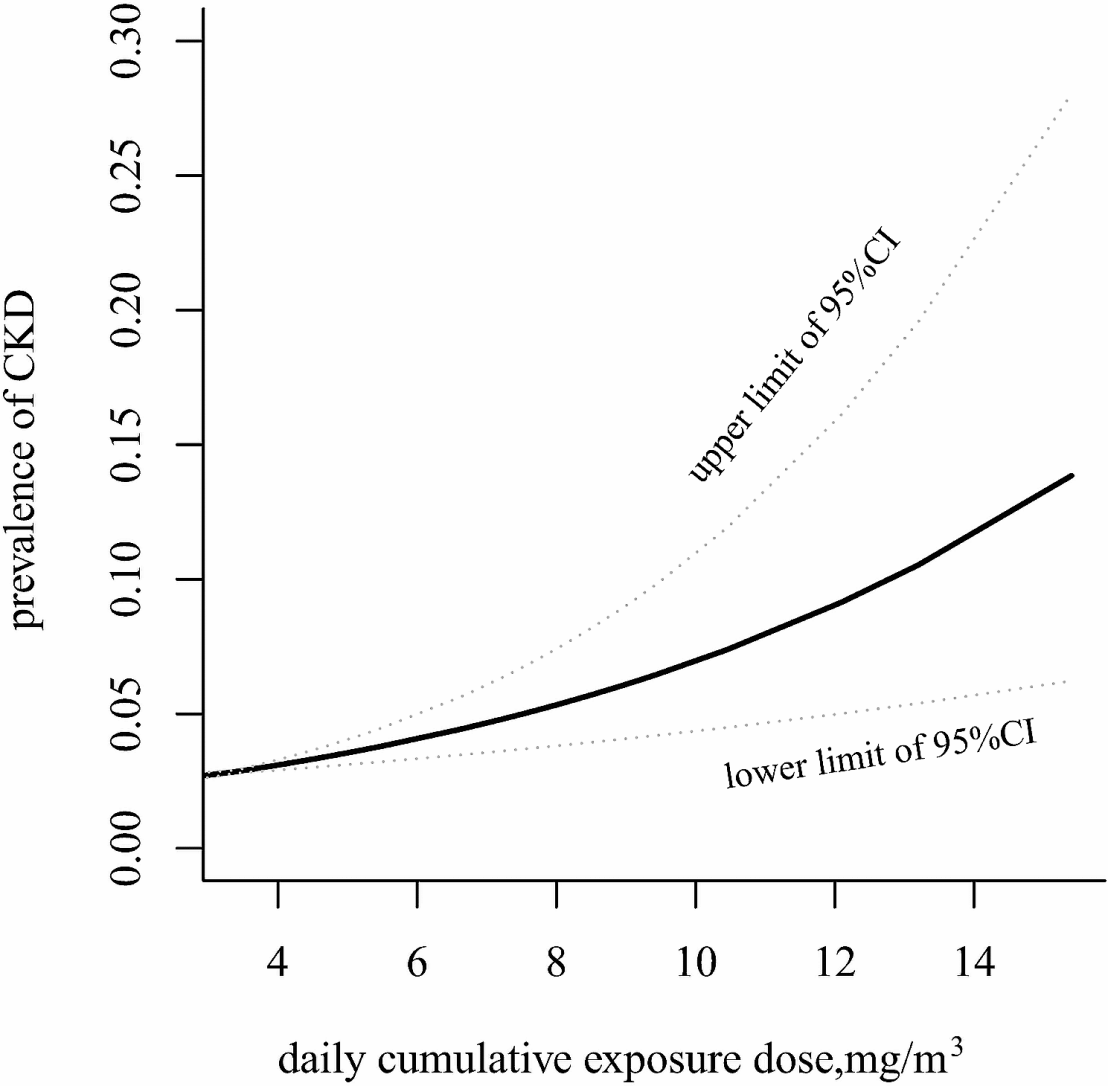
Smoothed curve fitting shows the relationship of MWF aerosols and CKD

The association of cumulative exposure to MWF aerosols and CKD prevalence using spline smoothing fittings is shown in Fig. 1. The prevalence of CKD showed a nonlinear relationship with cumulative MWF aerosols exposure. The threshold effect analysis showed that when the cumulative exposure dose exceeded the threshold value of 8.03 mg/m^3^-day, MWF aerosols exposure was associated with CKD (*OR* = 2.58, 95% *CI*: 1.30–5.12); after adjusting for confounders, the *OR* (95% *CI*) was 1.24 (95% *CI*: 1.03–1.58) (Table 4).

**Table 4:** Segmented logistic regression of cumulative MWF aerosols exposure, L_EX,8 h_, and prevalence of CKD

Variables	Model 1		Model 2	
	OR	(95% *CI*)	OR	(95% *CI*)
MWF aerosols exposure (mg/m^3^-day)				
0–8.03	1.00		1.00	
≥ 8.03	2.58	(1.30–5.12) ^*^	1.24	(1.03–1.58) ^*^
L_EX_,_8 h_ (dB(A))				
0–87.22	1.00		1.00	
≥ 87.22	1.99	(1.26–3.17) ^*^	1.16	(1.04–1.20) ^*^

Model 1: unadjusted; Model 2: Adjusted for physical exercise, smoking and drinking; ^*^P < 0.05

**Fig. 2:**
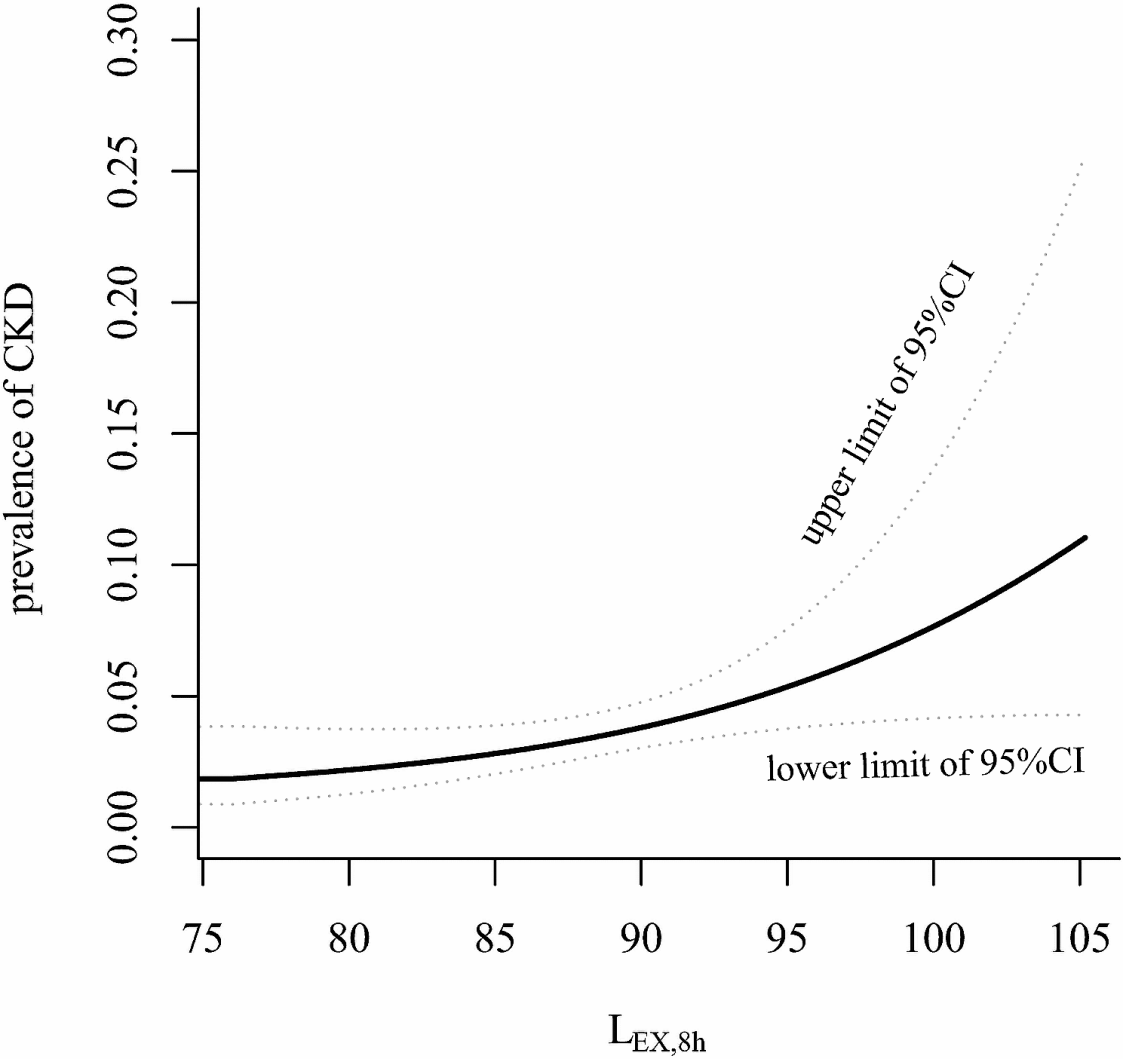
Smoothed curve fitting shows the relationship of L_EX,8 h_ and CKD

The relationship between occupational noise and the prevalence of CKD is shown in Fig. 2. When the L_EX,8 h_ exceeded the threshold value of 87.22 dB(A), the prevalence of CKD showed a nonlinear relationship with occupational noise exposure. Workers with L_EX,8 h_ ≥ 87.22 dB(A) had a higher prevalence of CKD than those with L_EX,8 h_ < 87.22 dB(A) (*OR* = 1.99, 95% *CI*: 1.26–3.17); after adjusting for confounders, the *OR* (95% *CI*) was 1.16 (95% *CI*: 1.04–1.20).

As shown in Table 5, we found a positive interaction between occupational MWF aerosols and noise exposure in the effect on the prevalence of CKD (*RERI* = 1.22, 95% *CI*: 0.16–3.21; *AP* = 0.45, 95% *CI*: 0.02–0.90; *S* = 3.54, 95% *CI*: 1.76–8.45). When the workers were exposed to both MWF aerosols cumulative exposure > 8.03 mg/m^3^ and L_EX,8 h_ > 87.22 dB(A), the proportion of the effect on the prevalence of CKD attributable to their interaction effect was 45%.

**Table 5:** Additive interactions between exposure to MWF aerosols, noise, and CKD

MWF aerosols (mg/m^3^-day)	Noise (dB(A))	OR (95% CI) ^a^	RERI (95% CI) ^a^	AP (95% CI) ^a^	S (95% CI) ^a^
< 8.03	< 87.22	1.00	1.22 (0.16–3.21)	0.45 (0.02–0.90)	3.54(1.76–8.45)
< 8.03	≥ 87.22	1.47 (1.18–1.85)			
≥ 8.03	< 87.22	1.10 (1.02–1.23)			
≥ 8.03	≥ 87.22	2.71 (1.48–4.96)			

^a^: adjusted for physical exercise, smoking and drinking

## Discussion

In the present cross-sectional study among machining workers, we found that both MWF aerosols and occupational noise were associated with a heightened risk of CKD. As the exposure dose to MWF and occupational noise increased, the prevalence of CKD was also escalated. Moreover, this study established that the daily cumulative exposure dose threshold of MWF aerosols for CKD was 8.03 mg/m^3^-day, and the threshold of noise exposure for CKD was 87.22 dB(A). We also showed that cumulative MWF aerosols and noise exposure had a positive interaction effect on CKD.

Our results showed that occupational MWF aerosols exposure may be associated with CKD, and the prevalence of CKD in workers rises with increasing cumulative exposure dose. This is the first epidemiological study to investigate the relationship of occupational MWF aerosols exposure and CKD. Previous animal experiments have confirmed that the presence of MCCPs and diethanolamine in MWF aerosols can cause kidney damage [37, 38]. In addition, PAHs in MWF aerosols activate the AhR signaling pathway, selectively damage glomerular cells, reduce GFR, gradually damage kidney tissue, and even lead to end-stage CKD [39]. MWF are often classified as straight (neat or mineral oils), soluble (a mixture of mineral and water-based), and synthetic (water-based, no oil) fluids [9]. Although we did not directly measure the harmful substances in MWF aerosols, according to previous studies, we may assume that the MWF of straight and soluble contains high concentrations of PAHs [40]. In vitro studies have shown that chronic persistent exposure to PAHs causes more serious glomerular damage than renal tubular damage [15, 16]. Mechanistically, the binding of PAHs to AhR in vivo preferentially depletes glutathione in glomerular mesangial cells compared with other ligands, interferes with glutathione homeostasis and mitochondrial function, and thus impairs renal cell function [41]. Moreover, the activation of AhR signaling pathway induces the upregulation of its target genes (including CYP1A1, CYP1A2, CYP1B1, and COX-2), reduces the expression of Mas receptor axis in the renin–angiotensin system (RAS), and increases reactive oxygen species (ROS) and oxidative stress, thereby inducing nephrotoxicity in the inflammatory process [42]. Our research findings indicate that there is a correlation between the daily cumulative exposure dose of MWF and an elevated risk of CKD among workers, which is in agreement with the conclusions drawn by Shrestha et al., who found that the hazard ratio for end-stage renal disease progressively rose in proportion to the increased cumulative exposure to straight MWF within the automotive worker cohort [11]. Diethanolamine is commonly added to the composition of MWF, functioning as a preservative. In a toxicological investigation assessing the impact of diethanolamine on mice, it was observed that an increase in the exposure dosage of diethanolamine corresponded with a rising incidence of renal tubular hyperplasia and the formation of renal tubular adenomas [43].

At present, there is no occupational exposure limit (OEL) of daily cumulative dose of MWF aerosols, and the threshold value calculated in this study (8.03 mg/m^3^-day) is higher than the recommended value (0.5 mg/m^3^, TWA) [8]. The NIOSH established the OEL for MWF aerosols at 0.5 mg/m^3^ to mitigate the potential risk associated with its exposure [8]. However, the absence of an OEL for water-soluble MWF, owing to their varying particle sizes, means that the aforementioned limit of 0.5 mg/m^3^ is applicable solely to straight MWF [9]. The MWF utilized in this study encompassed not only oil-based and water-based variants, making it challenging to determine the cumulative exposure from different types of MWF. Consequently, further research is imperative to provide guidance for the establishment of OELs specific to MWF.

In addition, our study shows that occupational exposure to noise increases the risk of CKD in workers, and an escalation in the intensity of noise exposure is accompanied by an increased risk of CKD in the workforce. Our findings align with two previous cross-sectional studies focused on the relationship between occupational noise and renal function. A cross-sectional study in South Korea showed a higher CKD prevalence in females who experienced long-term occupational noise (≥ 240 months) and a one-month increase in occupational noise exposure was associated with a decrease in eGFR in females aged < 60 years; however, the significant relationship was not found in male participants and female individuals aged between 60 and 79 years [44]. Furthermore, Chen et al. observed a positive correlation between cumulative noise exposure and mild renal impairment in petrochemical workers [45]. Lue et al. also found that noise was associated with a lower eGFR in B6C3F1 mice [46]. Prolonged exposure to noise induces a stress response that activates the sympathetic and endocrine systems, escalating oxidative stress, triggering pro-oxidase enzymes, and leading to an excess of ROS in the kidneys. The consequent oxidative stress results in the buildup of tyrosine-rich proteins, which in turn cause proteinuria and contribute to the progression of glomerular sclerosis and renal tubular interstitial fibrosis, thereby promoting the onset and progression of CKD [47]. The noise threshold of 87.22 dB(A) calculated in this study is slightly higher than the common critical value of 85 dB(A). Our findings show that the established permissible OELs are suitable for preventing noise-related renal damage.

Our results showed that there may be a positive interaction between MWF aerosols and noise in the development of CKD. Prior research has established that PAHs within MWF aerosols can activate the AhR, which may suppress the RAS and exacerbate the sympathetic and endocrine stress response induced by noise [42]. This activation can result in heightened oxidative stress and an overproduction of ROS within the kidneys, thereby augmenting the risk of renal impairment and the potential for development and progression of CKD. Our study suggests that joint effects of MWF aerosols and noise deserve more concerns, and additional research is needed to elucidate the mechanisms behind the effects of concurrent exposure to noise and MWF aerosols on renal function.

This cross-sectional study explored the relationship among occupational MWF aerosols exposure, occupational noise exposure, their concurrent exposure, and CKD for the first time, and analyzed MWF aerosols and occupational noise exposure thresholds. However, our research also has some limitations. First, cross-sectional studies cannot determine causal relationships and consider the sequence of events, and we need to design more rigorous studies to prove these relationships. Diet and genetic variants as confounding factors were not considered, which may have affected the results. The subjects were machining factory workers, and the population representation was not strong, so the results cannot be extrapolated to the entire occupation population. Second, the workers are also likely to be exposed to unpredictable levels of noise in their daily lives which may affect the assessment of occupational noise hazards; Due to the high cost and considerable effort involved in collecting individual occupational noise and MWF aerosols data, the individual sampling size is relatively small, which may influence the results. Finally, this study used the new CKD-EPI creatinine GFR equation developed in 2021 to evaluate renal function without considering ethnicity, which, in the study of the Asian population, may lead to a decrease in the prevalence of CKD and an overestimation of GFR values [48]. This requires researchers to consider including more Asian groups to correct the equation.

## Conclusions

In this cross-sectional study, occupational MWF aerosols and noise were positively and nonlinearly associated with CKD, and occupational MWF aerosols and noise exposure showed a positive interaction with CKD. Our findings preliminarily revealed that MWF aerosols and occupational noise might be a risk factor for reduced renal function. Prospective and longitudinal cohort studies are also necessary to elucidate the causality of these associations.

### Electronic supplementary material

Below is the link to the electronic supplementary material.

Additional file 1: Table S1: Comparison of general demographic characteristics of responders and nonresponders

## Data Availability

The datasets generated during and/or analyzed during the current study are not publicly available due to personal privacy and hospital requirements that employee medical examination data cannot be disclosed, but are available from the corresponding author on reasonable request.

## Abbreviations

CKD: Chronic kidney disease
NIOSH: The National Institute for Occupational Safety and Health
MWF: Metalworking fluids
MCCPs: Medium chain chlorinated paraffins
PAHs: Polycyclic aromatic hydrocarbons
AhR: Aryl hydrocarbon receptor
CNC: Numerical control machining center
8-hr: TWA 8-hour time weighted average allowable concentration
GFR: Glomerular filtration rate
UACR: Urinary albumin/creatinine ratio
CKD-EPI: Chronic Kidney Disease Epidemiology Collaboration
BMI: Body mass index
T2DM: Type 2 diabetes mellitus
RERI: Relative excess risk caused by interaction
AP: Attributable proportion due to interaction
S: Synergy index
OR: Odds ratio
CI: Confidence interval
RAS: Renin-angiotensin system
ROS: Increases reactive oxygen species
OEL: Occupational Exposure Limit
