# Supplementary material for: Association between exposure to metalworking fluid aerosols, occupational noise and chronic kidney disease: a cross-sectional study in China
Source: BMC Public Health. 2024 Jun 4;24:1495. doi: 10.1186/s12889-024-19006-7 (PMC11151621; doi:10.1186/s12889-024-19006-7)
Supplement: Supplementary file 1 — Additional file 1: Table S1: Comparison of general demographic characteristics of responders and nonresponders [file 12889_2024_19006_MOESM1_ESM.docx]

Additional file 1: Table S1 Comparison of general demographic characteristics of responders and nonresponders

| Variable | Responders (2738) | Nonresponders (92) | *P* |
| --- | --- | --- | --- |
| Sex |  |  | 0.285 |
| Male | 2,280 | 73 |  |
| Female | 450 | 19 |  |
| Age |  |  | 0.998 |
| < 30 | 854 | 29 |  |
| 30–40 | 1,023 | 34 |  |
| 40–50 | 578 | 19 |  |
| ≥ 50 | 283 | 10 |  |
| Nation |  |  | 0.104 |
| Han | 2,719 | 90 |  |
| Minority | 19 | 2 |  |
| Marital status |  |  | 0.925 |
| Unmarried | 752 | 25 |  |
| Married | 1,966 | 66 |  |
| Divorced | 20 | 1 |  |
| BMI |  |  | 0.362 |
| < 24 mg/m^2^ | 2,530 | 85 |  |
| ≥ 24 mg/m^2^ | 208 | 7 |  |
| Physical exercise |  |  | 0.542 |
| High | 346 | 9 |  |
| Moderate | 596 | 16 |  |
| Low | 1,796 | 67 |  |
| Smoking |  |  | 0.968 |
| Never | 1,526 | 51 |  |
| Occasionally | 526 | 17 |  |
| Frequently | 686 | 24 |  |
| Drinking |  |  | 0.977 |
| Never | 1,695 | 57 |  |
| Occasionally | 1,019 | 34 |  |
| Frequently | 24 | 1 |  |
| Use of protective equipment |  |  | 0.203 |
| Yes | 2,624 | 88 |  |
| No | 114 | 4 |  |
| Length of service |  |  | 0.205 |
| 1–5 years | 2,337 | 78 |  |
| 5–10 years | 250 | 8 |  |
| ≥ 10 years | 151 | 6 |  |
| Family history of CKD |  |  | 0.626 |
| No | 2,696 | 90 |  |
| Yes | 42 | 2 |  |
